# A Giga-Stable Oscillator with Hidden and Self-Excited Attractors: A Megastable Oscillator Forced by His Twin

**DOI:** 10.3390/e21050535

**Published:** 2019-05-25

**Authors:** Thoai Phu Vo, Yeganeh Shaverdi, Abdul Jalil M. Khalaf, Fawaz E. Alsaadi, Tasawar Hayat, Viet-Thanh Pham

**Affiliations:** 1 Faculty of Electrical and Electronics Engineering, Ton Duc Thang University, Ho Chi Minh City, Vietnam; 2 Biomedical Engineering Department, Amirkabir University of Technology, Tehran 15875-4413, Iran; 3 Ministry of Higher Education and Scientific Research, Baghdad 10045, Iraq; 4 Department of Information Technology, Faculty of Computing and IT, King Abdulaziz University, Jeddah 21589, Saudi Arabia; 5 Department of Mathematics, Quaid-I-Azam University, Islamabad 45320, Pakistan; 6 NAAM Research Group, King Abdulaziz University, Jeddah 21589, Saudi Arabia; 7 Nonlinear Systems and Applications, Faculty of Electrical and Electronics Engineering, Ton Duc Thang University, Ho Chi Minh City, Vietnam

**Keywords:** chaotic oscillators, megastability, hidden attractors, entropy

## Abstract

In this paper, inspired by a newly proposed two-dimensional nonlinear oscillator with an infinite number of coexisting attractors, a modified nonlinear oscillator is proposed. The original system has an exciting feature of having layer–layer coexisting attractors. One of these attractors is self-excited while the rest are hidden. By forcing this system with its twin, a new four-dimensional nonlinear system is obtained which has an infinite number of coexisting torus attractors, strange attractors, and limit cycle attractors. The entropy, energy, and homogeneity of attractors’ images and their basin of attractions are calculated and reported, which showed an increase in the complexity of attractors when changing the bifurcation parameters.

## 1. Introduction

In dynamic systems, there exists a type of categorization, which divides these systems into two groups: the first group includes systems which have self-exited attractors, and the second group includes systems with hidden attractors [1,2]. Self-exited attractor means that at least one equilibrium can be observed in its basin of attraction [3]. If there is no equilibrium in an attractor’s basin of attraction, that attractor is a hidden attractor.

Designing new special chaotic systems is a hot topic in the literature. There are chaotic systems which have no equilibria [4,5,6,7]. Some special systems have only stable equilibria [8,9] or lines of equilibria [10]. Similarly there are some systems with surfaces [11,12] and curves of equilibria [13,14,15], with multi-scroll attractors [16,17,18,19], fractional-order [20,21,22,23], free control [24,25], with non-hyperbolic equilibria [26], with offset boosting, having hyperchaotic attractors [27,28], conditional symmetry [29], and with real-world applications [30,31].

Multi-stability is one of the critical topics in dynamical systems [32,33]. This phenomenon has some advantages and disadvantages in different cases. For example, it is useful for allowing flexibility in the system’s performance without changing parameters. However, multi-stability is unfavorable in designing some commercial devices that should work in a noisy environment. Exceptional cases of multi-stability are extreme multi-stability [34,35,36] and megastability [37,38,39,40,41]. Systems with extreme multi-stability have an infinite uncountable number of coexisting attractors [42], while systems with megastability have an infinite countable number of coexisting attractors [43,44,45].

Feature extraction from images is an essential term for accurately analyzing them. Analysis of a large amount of data requires a lot of memory, computation, and cost. Feature extraction solves these problems by specifying the data with enough correctness [46]. The texture is a vital feature utilized to recognize objects or sections of interest in an image. It comprises essential information from the structure of images [47].Texture analysis is a general method for feature extracting from images. One of the most popular methods for extracting texture features is the Gray Level Co-occurrence Matrix (GLCM) that was introduced by Haralick et al. for the first time [48]. GLCM is a two-dimensional matrix that is $p_{i,j}\left(d,\theta \right)$ of $i^{th}$ and $j^{th}$ pixels with distance d in four directions according to θ that can be 0°, 45°, 90° and 135° [49,50]. Some statistical measures can be computed using GLCM such as energy, contrast, entropy, correlation, homogeneity, etc. [50,51]. 

In this paper, by modifying the system mentioned in [37], we design a four-dimensional system with unique features mentioned in the following sections. This system also has chaotic solutions and infinite hidden attractors. The regularity of the system’s attractors in the limit cycle, torus, and chaotic modes are compared with each other by calculating the GLCM matrix of their trajectories and the basins of attraction’s images and then extracting the entropy, energy, and homogeneity of these images.

In the next section, the designed system is introduced, and then thoroughly investigated in Section 3. The numerical stability analysis of the proposed system is investigated in Section 4, and in Section 5 the attractors with basins of attraction are given. The image processing and the features of attractors’ images are done in Section 6, and we conclude in Section 7.

## 2. The Main System

Consider the system below given by Kahn et al. [52], and modified by Sprott et al. in [37]:

(1) $$\dot{x}=y\;\dot{y}=-k^{2}x+\;ycos\left(x\right)$$

**Figure 1:**
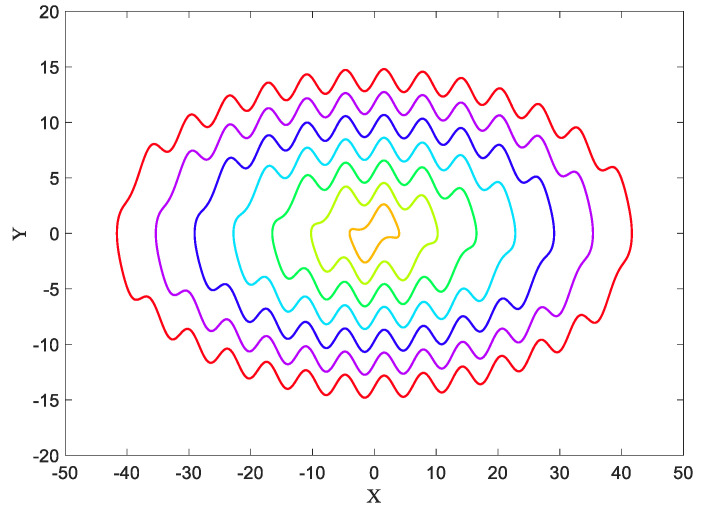
Hidden attractors of System (1) with seven different initial conditions. Except for the inner limit cycle, the rest of the attractors are hidden attractors.

This system consists of infinite layer–layer limit-cycles, which are hidden, except for the inner one. By giving different initial conditions to this system, hidden attractors appear. In Figure 1 seven attractors of System (1) are shown. The parameter k is set to k=0.33. Since the equilibrium point of this system is (0,0), and this point is unstable, the nearest limit cycle to this point is the self-excited attractor. However, the other limit cycles are hidden attractors. The attractors were plotted with initial conditions ((2n−1)π,0) that n=1,2,…,7.

**Figure 2:**
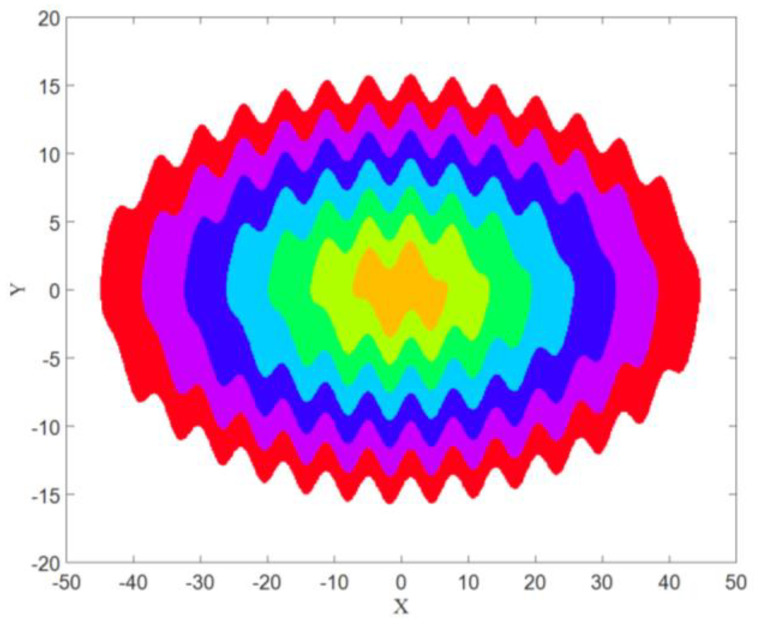
The basins of attraction of System (1) with values of parameters k=0.33.

The basins of attractions of limit cycles that are shown in Figure 1, and can be seen in Figure 2 with the same colors as the attractors. It has been obtained using a mesh of 500 × 500 initial conditions (*x* = −50:0.2:+50, and *y* = −20:0.08:+20). 

## 3. The Proposed System by Coupling Two Oscillators

We aim to design a system inspired by the system mentioned in the previous section. In this system, two oscillators of the form of Equation (1) were coupled one way. Therefore, we generated the following system, and the goal was to investigate particular characteristics of this system and find possible chaotic solutions.

(2) $$\dot{x}=y\;\dot{y}=-k^{2}x+\;ycos\left(x\right)+Az\;\dot{z}=\omega u\;\dot{u}=\omega \left(-k^{2}z+\;ucos\left(z\right)\right)$$

**Figure 3:**
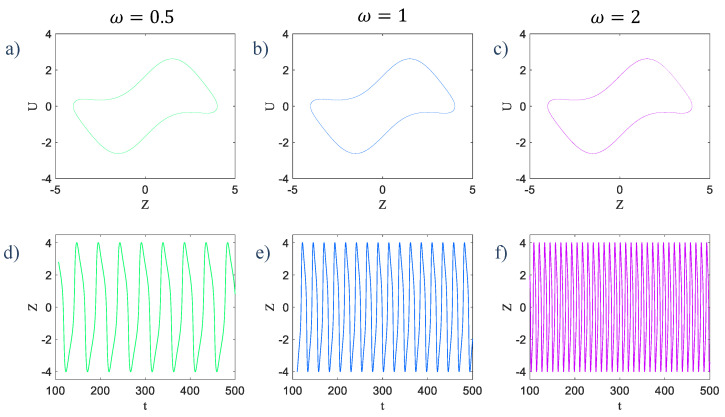
(**a**) The limit cycle of z and u variables in System (3) with ω=0.5, (**b**) ω=1, and (**c**) ω=2 that are the same attractors and changing the parameter ω has no effect on the attractor’s topology, (**d**) The time series of variable z in ω=0.5, (**e**) ω=1, and (**f**) ω=2 that differ with each other in proportion to the value of ω as the frequency tuner.

The second half of these equations does not depend on the first half, but affects it. The first two equations are the main equations in System (1), with the difference that the z variable is added to the second equation as a forcing term. The third and fourth equations are similar to those of our primary system (or System (1)), with a multiplying term ω, which tune the frequency of oscillations. Consider the second part of System (2) as an independent system. By giving three different values for ω and setting k to 0.33, it can be seen that the limit cycle attractors are the same but time series rely on the value of ω. Figure 3 shows the time series and limit cycles of this sub-system with ω=0.5, 1, and 2, respectively.

## 4. Numerical Stability Analysis

The only fixed point of the System (2) is [0, 0, 0, 0] and the Jacobian matrix is:

(3) $$\;J=\left[\begin{array}{c} 0\\ -k^{2}-ysin\left(x\right)\\ 0\\ 0 \end{array}\begin{array}{c} 1\\ \cos\left(x\right)\\ 0\\ 0 \end{array}\begin{array}{c} 0\\ A\\ 0\\ -\omega k^{2}-\omega usin\left(z\right) \end{array}\begin{array}{c} 0\\ 0\\ \omega \\ \omega cos\left(z\right) \end{array}\right]\overset{\left(x,y,z,u\right)=\left(0,0,0,0\right)}{\Rightarrow }\;J=\left[\begin{array}{c} 0\\ -k^{2}\\ 0\\ 0 \end{array}\begin{array}{c} 1\\ 1\\ 0\\ 0 \end{array}\begin{array}{c} 0\\ A\\ 0\\ -\omega k^{2} \end{array}\begin{array}{c} 0\\ 0\\ \omega \\ \omega \end{array}\right]$$

therefore, the eigenvalues are:

(4) $$\left|\lambda I-J\right|=0\;\to \left|\begin{array}{c} \lambda \\ k^{2}\\ 0\\ 0 \end{array}\begin{array}{c} -1\\ \lambda -1\\ 0\\ 0 \end{array}\begin{array}{c} 0\\ -A\\ \lambda \\ \omega k^{2} \end{array}\begin{array}{c} 0\\ 0\\ -\omega \\ \lambda -\omega \end{array}\right|=0\;\to \;\lambda^{4}-\lambda^{3}\left(\omega +1\right)+\lambda^{2}(\omega +\;k^{2}\omega^{2}+k^{2})-\lambda (k^{2}\omega^{2}+k^{2}\omega )+k^{4}\omega^{2}=0\;\to \lambda_{1,2}=\frac{\omega }{2}\left(1\pm \sqrt{-\left(2k-1\right)\left(2k+1\right)}\right)=\frac{\omega }{2}\left(1\pm \sqrt{1-4k^{2}}\right),\;\lambda_{3,4}=\;\frac{1}{2}\left(1\pm \sqrt{-\left(2k-1\right)\left(2k+1\right)}\right)=\frac{1}{2}\left(1\pm \sqrt{1-4k^{2}}\right)$$

It can be seen that this equilibrium point is unstable in any parameter value.

**Figure 4:**
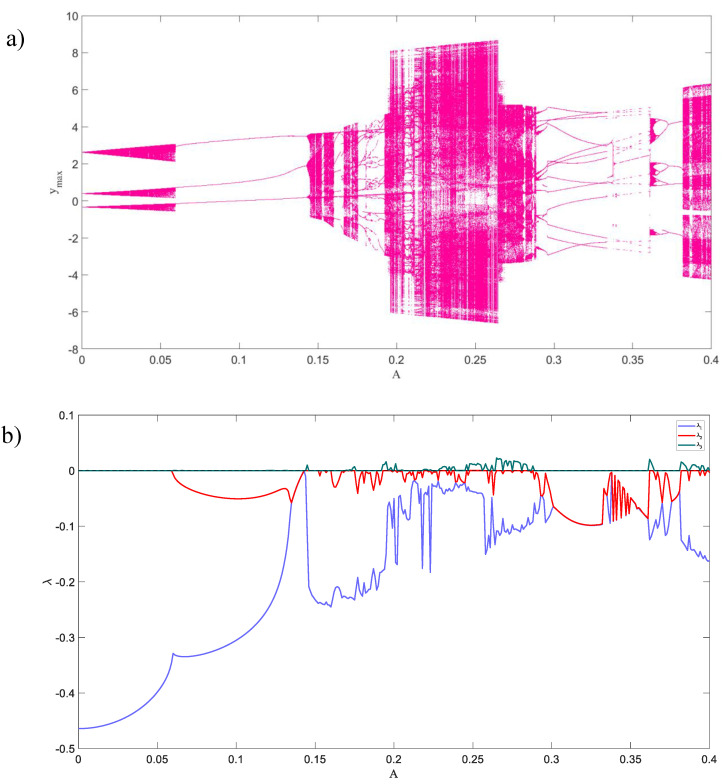
(**a**) The bifurcation diagram of the System (3), and (**b**) the Lyapunov exponents of the System (3).

The parameters of System (2) are chosen by trial and error in such a way that we detect chaotic solution. By selecting k=0.33 and ω=2.77, we choose parameter A as the bifurcation parameter. Figure 4 shows the bifurcation diagram and Lyapunov exponents’ spectrum versus A, for the inner attractor. For A<0.06, the dynamic is an attracting torus because of two zero and one negative Lyapunov exponents [53] and in larger values of A periodic and chaotic attractors can be seen.

## 5. The Attractors and Their Basins of Attraction

**Figure 5:**
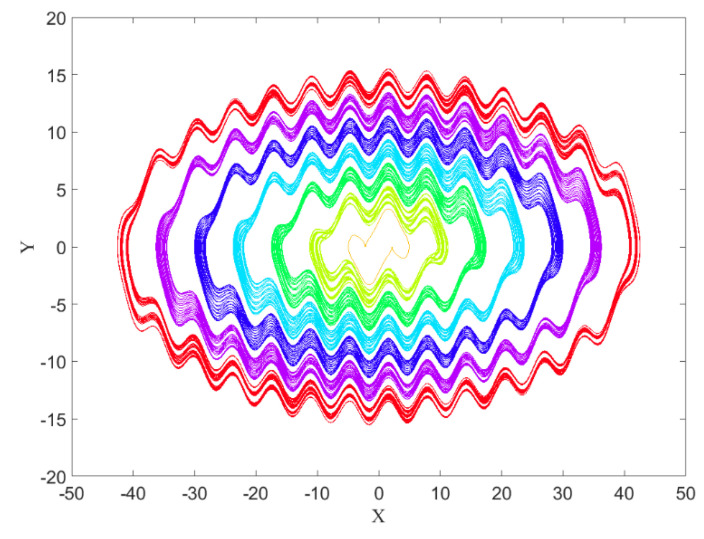
The self-excited attractor (inner limit cycle) and six hidden torus attractors of System (2) with different initial conditions and value of parameter A=0.1.

Choosing A=0.1, we obtain Figure 5 with seven different initial conditions ((0.1, 0.1, 0.1, 0.1), (3, 3, 0.1, 0.1), (4, 4, 0.1, 0.1), (6, 6, 0.1, 0.1), (9, 9, 0.1, 0.1), (10, 10, 0.1, 0.1) and (12, 12, 0.1, 0.1)). Except for the inner limit cycle that is self-excited, others are hidden torus attractors. 

**Figure 6:**
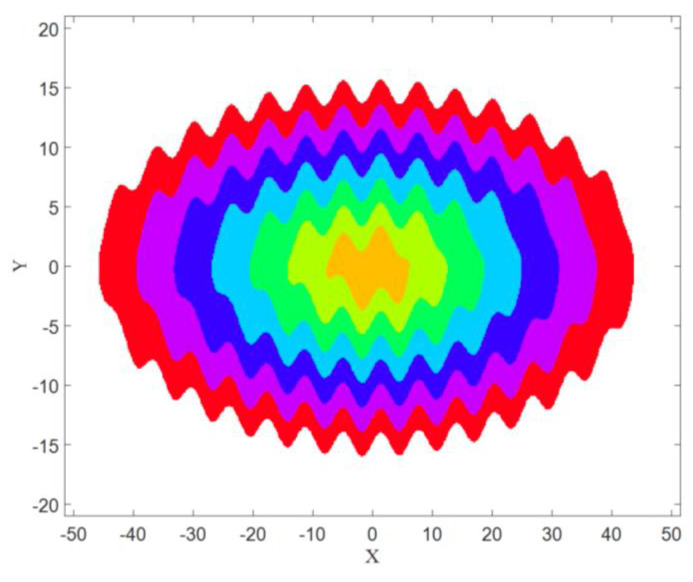
The basin of attraction of the attractors shown in Figure 5 with A=0.1.

The basin of attraction for each attractor is shown in Figure 6. The color of each basin was the same color as the corresponding attractor (from Figure 5). It was obtained using a mesh of 500 × 500 initial conditions (*x* = −50:0.2:+50, and *y* = −20:0.08:+20). 

**Figure 7:**
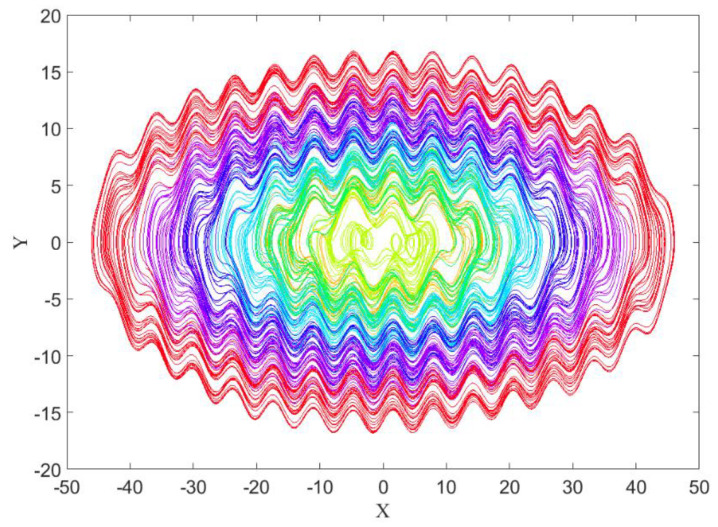
The self-excited attractor (inner strange attractor) and other hidden attractors of System (3) with different initial conditions for A=0.25.

By further changing parameter A to 0.25, the chaotic solution was observed. The first seven attractors are plotted in Figure 7 with the same initial conditions as before.

**Figure 8:**
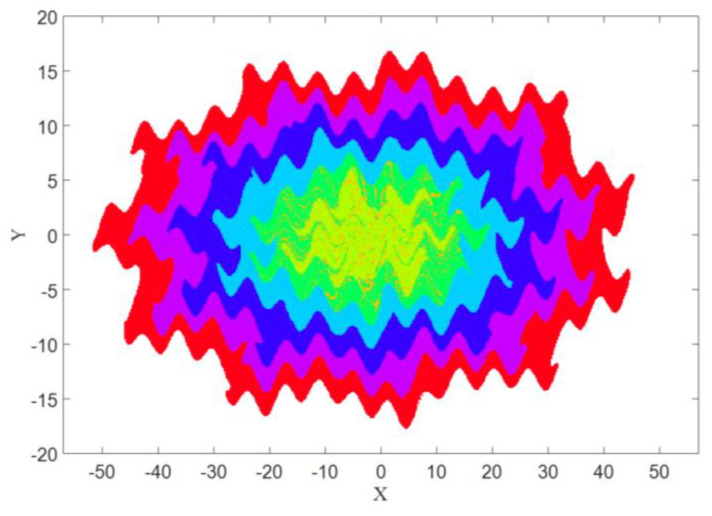
Basin of attraction of attractors shown in Figure 7 with A=0.25 with a chaotic solution.

Figure 8 shows the corresponding basins of attraction for the chaotic attractors from Figure 7. This figure shows that the first, second, and third basins are intertwined together. It was obtained using a mesh of 500 × 500 initial conditions (*x* = −50:0.2:+50, and *y* = −20:0.08:+20). 

## 6. Entropy Analysis

**Figure 9:**
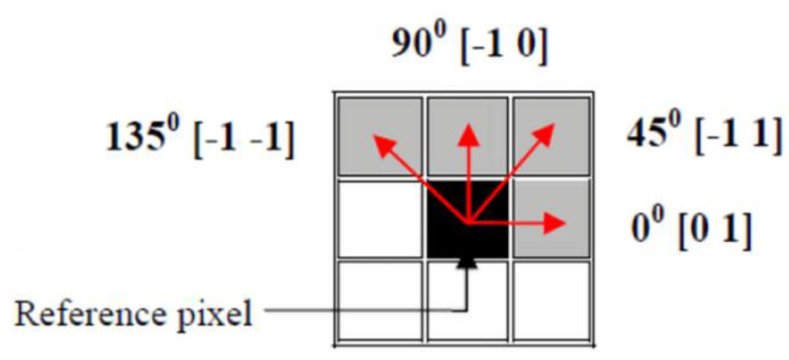
The GLCM matrix with different directions and angles.

As mentioned before, texture analysis is a popular method for image feature extraction. Important structural form of plane information is in the texture. The GLCM method is a method for extracting second-order statistic features of textures. The definition of GLCM is that it is a two dimensional histogram of gray levels for two pixels that are parted by certain spatial correlation. In other words GLCM is a matrix that shows the relative frequency of two pixels by using a displacement vector and the angle between them. GLCM matrix elements are the second order probability values that show changes between gray levels on i and j pixels of the image at a particular displacement distance d with a particular angle θ. The usual values of the angle are 0°,45°,90°, and 135° which are shown in Figure 9, and the GLCM matrix can be calculated separately at each angle. The default value of the displacement vector is equal to 1. GLCM have been used in many applications [54,55,56,57,58]. The exact calculation of the GLCM matrix is described in the literature [46,49]. Therefore, the aim of the rest of this research is the calculation of entropy, energy, and homogeneity from GLCM matrix. 

One of the important measures that indicate the level of complexity of an image is called Entropy. This feature is higher in the images that are not a smooth image in terms of texture [47]. Thus, the more complex and chaotic the image, the larger the value of entropy. Entropy is calculated with the GLCM matrix elements through the following formula:

(5) $$Entropy=-\sum_{i=0}^{n-1}\sum_{j=0}^{n-1}p_{ij}\log p_{ij}$$

where $p_{ij}$ is the element of GLCM matrix.

The second statistical measure that is known as Angular second moment, is Energy. This measure also detects irregularity in the texture of images and the difference of it from Entropy is that the value of Energy becomes larger in smooth images when the gray level of images is steady or periodic states. Thus Energy has an inverse relationship with Entropy. 

The formulation of energy is:

(6) $$Energy=\sum_{i=0}^{n-1}\sum_{j=0}^{n-1}p_{ij}^{2}$$

$p_{ij}$ is similarly the element of GLCM matrix. The normalized range of Energy is [0,1]. This measure is equal to 1 in a constant image.

Another important feature is Homogeneity, which is called Inverse Difference Moment and measures the homogeneity of the image. The maximum value of this measure occurs when all elements of the image are equal. 

The equation of Homogeneity is:

(7) $$Homogeneity=\sum_{i=0}^{n-1}\sum_{j=0}^{n-1}\frac{1}{1+{\left(i-j\right)}^{2}}p_{ij}$$

the range of Homogeneity is also [0,1] and reaches 1 for a diagonal GLCM.

Now the goal is calculating these three features in four different angles for Figure 1, Figure 5 and Figure 7, then for the basins of attraction that are Figure 2, Figure 6 and Figure 8.

### 6.1. The Entropy of Attractors

**Figure 10:**
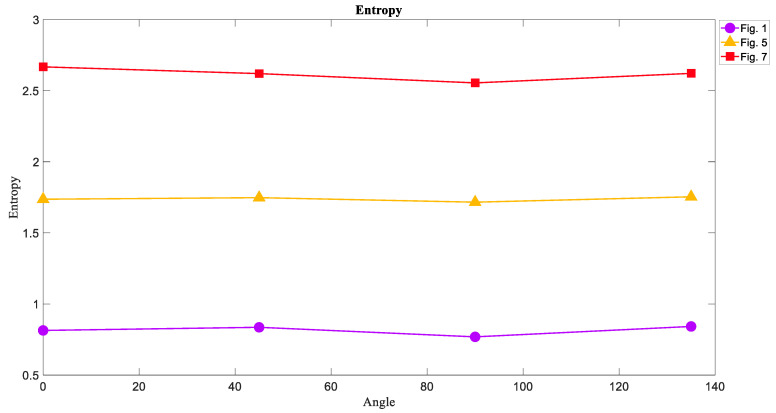
The Entropy measure of Figure 1, Figure 5, and Figure 7 in four directions 0°, 45°, 90°, and 135°. The Entropy of chaotic attractors is larger than that of torus and limit cycle attractors.

The results of calculating the entropy of attractor images in four directions are shown in Figure 10. As expected, the Entropy of chaotic attractors shown in Figure 7 had the highest value in each direction because of more irregularity than others in its texture. Then, Figure 5 including torus attractors in yellow had the middle value of Entropy, and the limit cycle attractors in Figure 1 were on the lower level of Entropy. This result proves that the Entropy of chaotic attractor images is higher than that of regular attractors.

### 6.2. The Energy of Attractors

**Figure 11:**
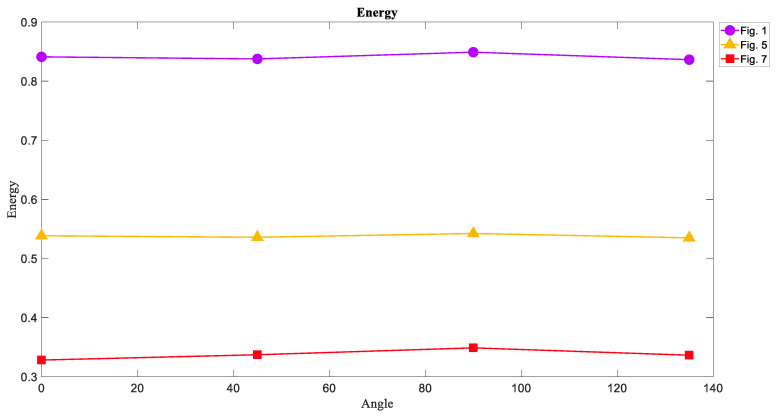
The Energy measure of Figure 1, Figure 5, and Figure 7 in four directions 0°, 45°, 90°, and 135°. The Energy of the limit cycle attractors is larger than the Energy of the torus and chaotic attractors contrary to Entropy.

The Energy of images has a reverse correlation with Entropy. In Figure 11 it can be seen that the Energy of chaotic attractors had the lowest value in each angle and the limit cycle attractors were on ahigh level of Energy. Given the inverse relationship between Entropy and Energy, one can expect that the chaotic attractor is on the lowest level of Energy compared to the other attractors.

### 6.3. The Homogeneity of Attractors

**Figure 12:**
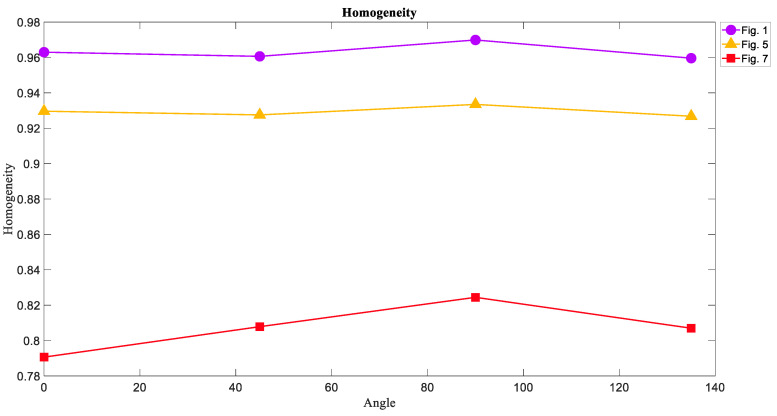
The Homogeneity measure of Figure 1, Figure 5, and Figure 7 in four directions 0°, 45°, 90°, and 135°. The Homogeneity of the limit cycle attractors is larger than the Homogeneity of the torus and chaotic attractors that is the same result for Energy.

The results of the Homogeneity measure weresimilar to those of the Energy measure so that the most homogeneous texture wasfor Figure 1, and the Homogeneity of Figure 7 was the lowest in these three textures. Figure 12 showedthe values of Homogeneity in each direction for each image.

In all features, there is not a significant difference between each angle. 

### 6.4. The Entropy of Basins of Attraction

**Figure 13:**
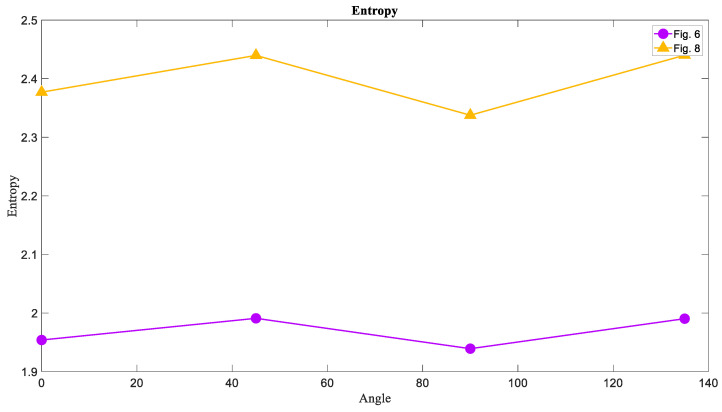
The Entropy measure of Figure 6 and Figure 8 in four directions 0°, 45°, 90°, and 135°. The Entropy of the limit cycle basins is lower than the Entropy of chaotic basins of attraction.

Now the calculation of three measures is desired for the basins of attraction images that are Figure 2, Figure 6, and Figure 8. Since the basins of System (1) and System (2) in the limit cycle mode were almost the same, the comparison was between Figure 6 and Figure 8. As predicted before, the chaotic basin has more amount of Entropy than the limit cycle basin. Figure 13 showed the Entropy values of Figure 6 and Figure 8 in four directions.

### 6.5. The Energy of Basins of Attraction

**Figure 14:**
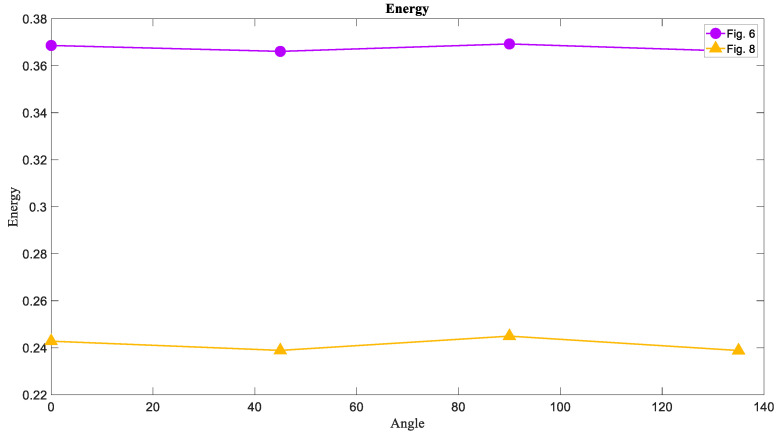
The Energy measure of Figure 6 and Figure 8 in four directions 0°, 45°, 90°, and 135°. The Energy of the chaotic basins of attraction is lower than the Energy torus basins of attraction that is the opposite result for Entropy.

The Energy measure of Figure 6 and Figure 8 was calculated, and the result was in line with our expectations. Thus, the Energy of limit cycle basins is larger than the Energy of chaotic basins in each direction as it can be seen in Figure 14.

### 6.6. The Homogeneity of Basins of Attraction

**Figure 15:**
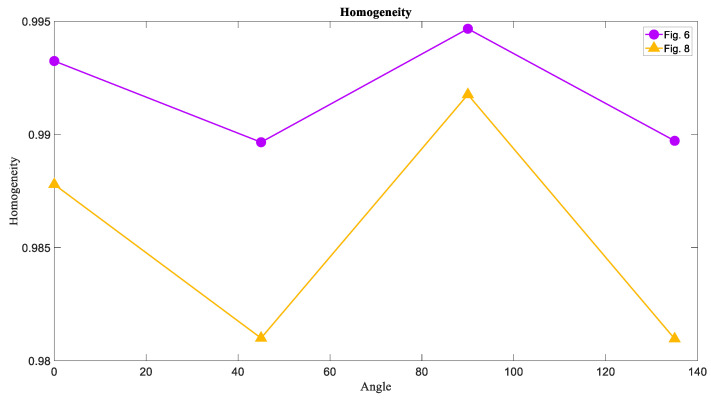
The Homogeneity measure of Figure 6 and Figure 8 in four directions 0°, 45°, 90°, and 135°. The Homogeneity of the chaotic basin of attraction is larger than the Homogeneity of torus basin of attraction that is the likewise result for Energy.

The last measure is the Homogeneity of Figure 6 and Figure 8 that was calculated and the results were similar to those of the Energy measure. Therefore, the Homogeneity of the chaotic basins that is Figure 8, is lower than the Homogeneity of the limit cycle and torus basins (Figure 6). The results are presented in a chart format in Figure 15.

## 7. Discussion and Conclusions

In this paper, we introduced a new four-dimensional nonlinear system that has infinite countable coexisting attractors. Those attractors appeared in an unusual topology. We described that oneof the attractors is self-excited and others are hidden. By modifying and choosing the parameters, we designed a complex system, which can have coexisting torus, strange attractors, and limit cycle attractor. We investigated the GLCM Entropy, Energy and Homogeneity on the figures of attractors and their basins and we concluded that the Entropy of chaotic behavior whether in the attractor or basin of attraction, is larger than the limit cycle or torus attractor. In contrast, the Energy and Homogeneity of the chaotic attractors are at the lowest level in comparison with torus and limit cycle attractors.
